# Fetal alleles predisposing to metabolically favorable adiposity are associated with higher birth weight

**DOI:** 10.1093/hmg/ddab356

**Published:** 2021-12-13

**Authors:** William D Thompson, Robin N Beaumont, Alan Kuang, Nicole M Warrington, Yingjie Ji, Jessica Tyrrell, Andrew R Wood, Denise M Scholtens, Bridget A Knight, David M Evans, William L Lowe Jr, Gillian Santorelli, Raq Azad, Dan Mason, Andrew T Hattersley, Timothy M Frayling, Hanieh Yaghootkar, Maria Carolina Borges, Deborah A Lawlor, Rachel M Freathy

**Affiliations:** Institute of Biomedical and Clinical Science, University of Exeter Medical School, University of Exeter, Exeter EX2 5DW, UK; Institute of Biomedical and Clinical Science, University of Exeter Medical School, University of Exeter, Exeter EX2 5DW, UK; Department of Preventive Medicine, Northwestern University Feinberg School of Medicine, Chicago, IL 60611, USA; MRC Integrative Epidemiology Unit, University of Bristol, Bristol BS8 2BN, UK; University of Queensland Diamantina Institute, University of Queensland, Brisbane QLD 4102, Australia; Department of Public Health and Nursing, NTNU, K.G. Jebsen Center for Genetic Epidemiology, Norwegian University of Science and Technology, Postboks 8905, N-7491, Norway; Institute of Biomedical and Clinical Science, University of Exeter Medical School, University of Exeter, Exeter EX2 5DW, UK; Institute of Biomedical and Clinical Science, University of Exeter Medical School, University of Exeter, Exeter EX2 5DW, UK; Institute of Biomedical and Clinical Science, University of Exeter Medical School, University of Exeter, Exeter EX2 5DW, UK; Department of Preventive Medicine, Northwestern University Feinberg School of Medicine, Chicago, IL 60611, USA; NIHR Exeter Clinical Research Facility, Royal Devon and Exeter NHS Foundation Trust, Exeter EX2 5DW, UK; MRC Integrative Epidemiology Unit, University of Bristol, Bristol BS8 2BN, UK; University of Queensland Diamantina Institute, University of Queensland, Brisbane QLD 4102, Australia; Department of Medicine, Northwestern University Feinberg School of Medicine, Chicago, IL 60611, USA; Bradford Institute for Health Research, Bradford Royal Infirmary, Duckworth Lane, Bradford BD9 6RJ, UK; Department of Biochemistry, Bradford Royal Infirmary, Bradford BD9 6DA, UK; Bradford Institute for Health Research, Bradford Royal Infirmary, Duckworth Lane, Bradford BD9 6RJ, UK; Institute of Biomedical and Clinical Science, University of Exeter Medical School, University of Exeter, Exeter EX2 5DW, UK; Institute of Biomedical and Clinical Science, University of Exeter Medical School, University of Exeter, Exeter EX2 5DW, UK; Institute of Biomedical and Clinical Science, University of Exeter Medical School, University of Exeter, Exeter EX2 5DW, UK; MRC Integrative Epidemiology Unit, University of Bristol, Bristol BS8 2BN, UK; Population Health, Bristol Medical School, University of Bristol, Bristol BS8 2BN, UK; MRC Integrative Epidemiology Unit, University of Bristol, Bristol BS8 2BN, UK; Population Health, Bristol Medical School, University of Bristol, Bristol BS8 2BN, UK; Bristol NIHR Biomedical Research Centre, Bristol BS8 2BN, UK; Institute of Biomedical and Clinical Science, University of Exeter Medical School, University of Exeter, Exeter EX2 5DW, UK; MRC Integrative Epidemiology Unit, University of Bristol, Bristol BS8 2BN, UK

## Abstract

**Background:**

Higher birthweight is associated with higher adult body mass index (BMI). Alleles that predispose to greater adult adiposity might act in fetal life to increase fetal growth and birthweight. Whether there are fetal effects of recently identified adult metabolically favorable adiposity alleles on birthweight is unknown.

**Aim:**

We aimed to test the effect on birthweight of fetal genetic predisposition to higher metabolically favorable adult adiposity and compare that with the effect of fetal genetic predisposition to higher adult BMI.

**Methods:**

We used published genome wide association study data (*n* = upto 406 063) to estimate fetal effects on birthweight (adjusting for maternal genotype) of alleles known to raise metabolically favorable adult adiposity or BMI. We combined summary data across single nucleotide polymorphisms (SNPs) with random effects meta-analyses. We performed weighted linear regression of SNP-birthweight effects against SNP-adult adiposity effects to test for a dose-dependent association.

**Results:**

Fetal genetic predisposition to higher metabolically favorable adult adiposity and higher adult BMI were both associated with higher birthweight (3 g per effect allele (95% CI: 1–5) averaged over 14 SNPs; *P* = 0.002; 0.5 g per effect allele (95% CI: 0–1) averaged over 76 SNPs; *P* = 0.042, respectively). SNPs with greater effects on metabolically favorable adiposity tended to have greater effects on birthweight (*R*^2^ = 0.2912, *P* = 0.027). There was no dose-dependent association for BMI (*R*^2^ = −0.0019, *P* = 0.602).

**Conclusions:**

Fetal genetic predisposition to both higher adult metabolically favorable adiposity and BMI is associated with birthweight. Fetal effects of metabolically favorable adiposity-raising alleles on birthweight are modestly proportional to their effects on future adiposity, but those of BMI-raising alleles are not.

## Introduction

High birth weight (>4 kg), compared with average birth weight, is associated with an increased risk of higher adult body mass index (BMI) (1). Moreover, across its distribution, higher birth weight is associated with higher BMI between the ages of 18 and 20 years (2). The mechanisms underlying the association between higher birth weight and higher adult BMI are not fully understood, but one possible mechanism could be that the inheritance of genetic variants that influence postnatal body mass also influences fetal growth and hence birth weght.

Previous twin studies and genetic association studies have failed to find strong evidence of a shared genetic association between adult BMI and fetal growth (3–6). Recently, however, using a much larger population sample than previous studies, a positive genetic correlation between birth weight and adult BMI was detected (7). Subsequent studies have shown that a BMI polygenic risk score, derived from genetic variants reaching genome wide significance in a large genome wide study of BMI (8), is predictive of birth weight (the top 10% of BMI polygenic risk having a 60 g higher birth weight than the bottom 10%) (9), suggesting that some of the fetal genetic predisposition to higher adult adiposity may influence birth weight.

Several genetic variants have been identified where one allele is associated with higher adult adiposity, but lower metabolic risk (i.e. lower risk of type 2 diabetes and cardiovascular disease), so called ‘metabolically favorable adiposity’ alleles (10). Metabolically favorable adiposity alleles may have this effect because they are associated with higher adiposity in the more metabolically stable subcutaneous adipose tissue, and decreased fat deposition in the liver (10,11). It is plausible that if the higher fat mass and insulin sensitivity associated with a genetic predisposition to metabolically favorable adiposity are present during fetal development, this genetic predisposition will have a positive influence on birth weight, since fetal insulin is a key fetal growth factor (12).

The primary aim of this study was to test the hypothesis that fetal genetic predisposition to adult metabolically favorable or general adiposity (the latter indexed by BMI) influences birth weight. To the best of our knowledge this is the largest sample size used to date (*N* = 406 063) to test whether fetal genetic predisposition to higher adult BMI affects birth weight, and the first to test the effect of fetal genetic predisposition for higher metabolically favorable adult adiposity on birth weight. A secondary aim of this study was to test the effects of the same fetal genetic predisposition to higher metabolically favorable adult adiposity or BMI on other perinatal anthropometric traits (length, ponderal index, head circumference and skinfold thickness) and cord-blood markers (insulin, c-peptide, leptin and adiponectin). This secondary aim was exploratory, given the relatively small sample sizes that we have available (*N* = 9350). All of the fetal genetic associations tested were adjusted for maternal genotype effects (7). This was to ensure that we were assessing the influence of alleles inherited by the fetus, un-confounded by maternal genetic variation that influences fetal genetic variation, and might also influence fetal growth via the intrauterine environment (13).

## Results

### Fetal genetic variants that predispose to higher metabolically favorable adiposity or to higher BMI are associated with higher mean birth weight

**Figure 1:**
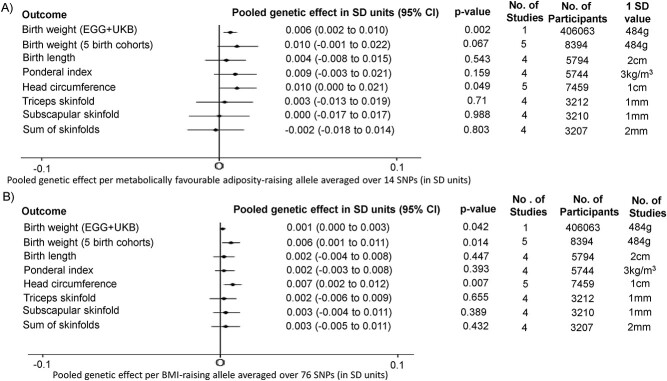
Pooled genetic effects of fetal (**A**) metabolically favorable adiposity and (**B**) BMI SNPs on birth anthropometric outcomes. (1) For birth weight and head circumference five studies are mentioned; this equates to ALSPAC, BiB, EFSOCH, HAPO 1 and HAPO 2. (2) For birth weight (EGG + UKB), the number of participants is the number involved in the GWAS of own birth weight adjusted for maternal genotype using the WLM (that is 101 541 UKB participants who reported their own birth weight and birth weight of their first child, 195 815 UKB and EGG participants with own birth weight data, and 108 707 UKB and EGG participants with offspring birth weight data) (7).

On average, alleles increasing metabolically favorable adiposity in adulthood were also related to heavier weight at birth (3 g (95% CI: 1–5) per effect allele; *P*-value = 0.0018; Fig. 1). This estimate was obtained by using random effects meta-analysis to pool single nucleotide polymorphism (SNP)-specific genetic associations with birth weight for 14 SNPs previously reported to be strongly associated with metabolically favorable adiposity in adults (10). Further details are presented in Materials and Methods; Main analyses: birth weight; Early Growth Genetics (EGG) + UK Biobank and Supplementary Methods.

On average, alleles increasing BMI in adulthood were also related to heavier weight at birth (0.5 g (95% CI: 0–1) per effect allele; *P*-value = 0.0422; Fig. 1). This estimate was obtained by using random effects meta-analysis to pool SNP-specific genetic associations with birth weight for 76 SNPs previously reported to be strongly associated with BMI in adults (8). Further details are presented in Materials and Methods; Main analyses: birth weight; EGG + UK Biobank and Supplementary Methods.

These results cannot be explained by confounding by the maternal genotype given all analyses controlled for this.

### The effect on birth weight of fetal genetic variants that predispose to higher metabolically favorable adiposity is proportional to their effect on adult adiposity

**Figure 2:**
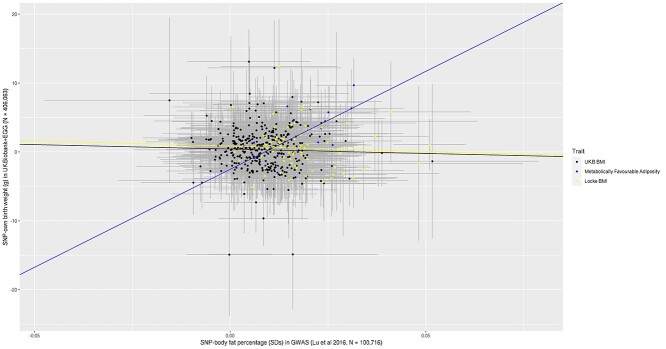
Scatter plot of 386 BMI SNPs identified in UK Biobank GWAS (15), 76 GIANT consortium SNPs (8) and 14 metabolically favorable adiposity SNPs (10) [SNP effects on body fat percentage (*x*-axis) and SNP effects on birth weight (*y*-axis)] to assess whether birth weight effects were proportional to adult adiposity effects. (1) We fitted a regression line to each set of SNPs that was weighted by the inverse of the standard errors of the SNP-birth weight associations. (2) The error bars represent the 95% confidence intervals. (3) The black line and data points represent the 386 BMI SNPs identified in a UK Biobank GWAS (15), the yellow line and data points represent the 76 GIANT consortium SNPs (8) and the blue line and data points represent the 14 metabolically favorable adiposity SNPs (10). (4) Of the 392 SNPs identified in both UK Biobank and Locke *et al*. (8), only 386 were available in Lu *et al*. (14).

**Figure 3:**
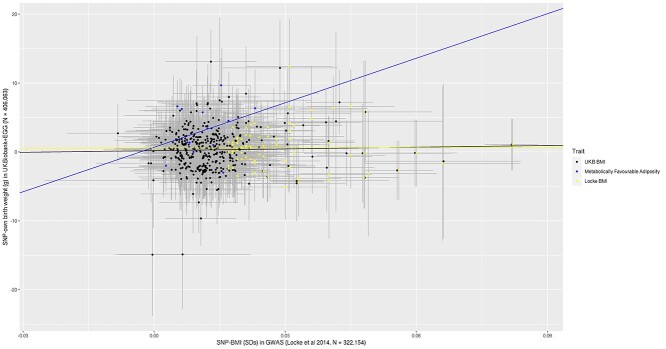
Scatter plot of 392 BMI SNPs identified in UK Biobank GWAS (15), 76 GIANT consortium SNPs (8) and 14 metabolically favorable adiposity SNPs (10) [SNP effects on BMI (*x*-axis) and SNP effects on birth weight (*y*-axis)] to assess whether birth weight effects were proportional to adult adiposity effects. (1) We fitted a regression line to each set of SNPs that was weighted by the inverse of the standard errors of the SNP-birth weight associations. (2) The error bars represent the 95% confidence intervals. (3) The black line and data points represent the 392 BMI SNPs identified in a UK Biobank GWAS (15), the yellow line and data points represent the 76 GIANT consortium SNPs (8) and the blue line and data points represent the 14 metabolically favorable adiposity SNPs (10).

To explore whether any effect on birth weight was proportionate to that on adult adiposity, we plotted the estimated SNP-birth weight effects against the estimated SNP-adult adiposity effects, measured either as body fat percentage (Fig. 2) or as BMI (Fig. 3). Weighted linear regression was used to fit the slope. To try and minimize the risk of overfitting, we extracted SNP-body fat percentage (14) and SNP–BMI (8) genetic association data from genome-wide association studies (GWASs) that did not include UK Biobank, since this study was a major contributor to the birth weight GWAS used in our analyses. The metabolically favorable adiposity alleles were associated with birth weight and adult measures of adiposity in a modest dose-dependent manner, with the alleles with the larger effects on body fat percentage having the larger effects on birth weight (*R*^2^ = 0.2912, *P*-value = 0.027). In contrast, there was no evidence that the BMI alleles were associated with birth weight and any adult measures of adiposity in a dose-dependent association (SNP-body fat percentage *R*^2^ = −0.0110, *P*-value = 0.670, SNP–BMI *R*^2^ = −0.0135, *P*-value = 0.971), suggesting the presence of substantial heterogeneity of effects on birth weight across BMI-associated SNPs.

### Effects in exploratory analyses of additional perinatal anthropometric outcomes were mostly directionally consistent with the main birth weight analysis

We undertook exploratory analyses of associations with additional anthropometric outcomes (birth weight (partially independent from EGG + UK Biobank), birth length, ponderal index, head circumference, triceps skinfolds, subscapular skinfolds and sum of skinfolds) from four pregnancy cohorts (*N* = 3207–8394). We considered these novel analyses to be exploratory because of relatively limited sample sizes available (details in Materials and Methods: Exploratory analyses: birth anthropometric measures and cord-blood outcomes). The effects of both fetal metabolically favorable adiposity and BMI pooled genetic effects on birth weight in the birth cohorts were consistent with the main (larger) analyses in EGG + UK Biobank (Fig. 1). Effects on birth length, ponderal index and head circumference pooled across these four cohorts were directionally consistent with the results for birth weight (Fig. 1).

**Figure 4:**
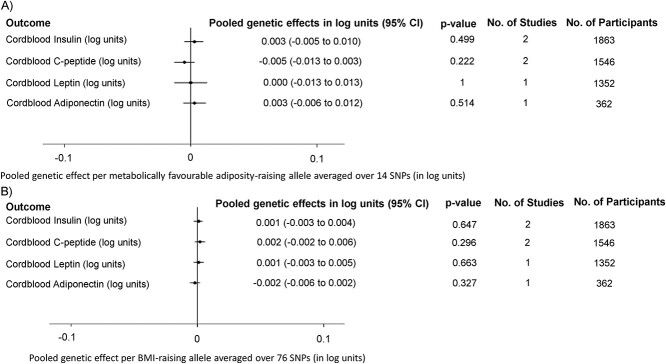
Pooled genetic effects of fetal (**A**) metabolically favorable adiposity and (**B**) BMI SNPs on cord-blood outcomes.

In three of the same four pregnancy cohorts we also undertook exploratory analyses with cord-blood measures of insulin, c-peptide, leptin and adiponectin (*N* = 362–1863) (details in Materials and Methods: Exploratory analyses: birth anthropometric measures and cord-blood outcomes). All of the fetal metabolically favorable adiposity or BMI pooled genetic effect estimates with these cord-blood markers were imprecise, with wide confidence intervals, making robust conclusions difficult (Fig. 4). There was little evidence of between study heterogeneity for any of the outcomes.

### Similar associations for fetal genetic predisposition to higher adult BMI observed using genetic variants identified in the UK Biobank

UK Biobank was a large contributor to both the metabolically favorable adiposity and birth weight GWAS’s, and therefore there is a possibility that our results might be biased by overfitting. Since there is no large scale GWAS for metabolically favorable adiposity that does not include UK Biobank, we investigated the possibility of such bias by repeating the BMI analyses with 392 SNPs selected from the UK Biobank (15). We checked to see whether the estimated genetic association of these 392 BMI SNPs was consistent with our main analyses where BMI SNPs were selected from a GWAS not including UK Biobank (8). Consistent with the main analyses, on average, alleles increasing BMI in adulthood were also related to heavier weight at birth (0.3 g (95% CI: 0–0.6) per effect allele, *P*-value = 0.0271). The scatter plots exploring proportionality was also similar to in these analyses to the main BMI analyses (SNP-body fat percentage *R*^2^ = −0.0013, *P*-value = 0.472; SNP–BMI *R*^2^ = −0.0019, *P*-value = 0.602; see Figs 2 and 3).

## Discussion

Using data on more than 400 000 individuals, we have shown that fetal genetic predisposition to higher adult metabolically favorable adiposity or BMI is associated with higher birth weight. The metabolically favorable adiposity SNPs had an effect on birth weight that was modestly proportional to that seen with adult adiposity (Fig. 2). In contrast, there was no strong correlation for the BMI SNPs between their effects on birth weight and adult adiposity (Fig. 3), highlighting heterogeneity of effects of fetal BMI genetic variants on fetal growth. The effect of the fetal genetic variants on other birth anthropometric outcomes was directionally consistent with their effects on birth weight. For cord-blood markers, effects of both metabolically favorable adiposity and BMI genetic variants were close to the null. However, we had limited power for results beyond birth weight and so these results should be treated with caution until replicated in larger studies.

This study provides further evidence of the contribution of fetal genetics relating to predisposition to higher adult adiposity in determining offspring birth weight. Early studies using BMI genetic variants, without adjusting for the maternal genotype, found little evidence of an effect on birth weight (5). This was corroborated by a GWAS of BMI during the early stages of childhood; the variants identified to be associated with BMI in early life had minimal effect on birth weight (16). However, a recent large scale GWAS, using LD score regression with a previous GWAS, found a small genetic correlation between BMI and fetal genetic effect on own birth weight, adjusted for maternal genotype (*r*_g_ = 0.12) (7). Our scatter plot analysis showed evidence of a dose response effect of metabolically favorable adiposity associated SNPs on birth weight, but this was not seen for BMI associated SNPs. These findings are consistent with the observation that most SNPs identified in the BMI GWAS map to genes expressed in the brain (8), in particular regions of the brain like the insula and substantia nigra that are implicated in reward and addiction processes (17). Therefore, these genetic variants might influence adult adiposity by influencing a neuronal pathway which in turn regulates postnatal appetite rather than general body growth.

This study, and previous research, provide further evidence of the interplay between maternal and fetal genetic effects on fetal growth. In a previous Mendelian randomization study, where we adjusted for the fetal genotype, we showed that higher maternal (genetically instrumented) metabolically favorable adiposity leads to lower offspring birth weight (18). Considering those findings with the ones from the current paper our work suggests that mothers with metabolically favorable adiposity alleles will on average have more metabolically favorable adiposity, and fetal intrauterine exposure to this would result in them having a lower birth weight. At the same time, the fetuses of these women will inherit more metabolically favorable adiposity alleles, compared with children of women without these alleles, which will result in them having higher birth weight. Completely separating the maternal from fetal effects may be difficult and highlights the importance for adjusting for maternal genetic variants as we have done here, and for the fetal genetic variants in Mendelian randomization studies of maternal pregnancy exposures as we have done previously (18). A recent GWAS of fetal genetic effects on own birth weight, adjusted for maternal genotype, found that genetic predisposition to higher adult fasting glucose levels was associated with lower birth weight, possibly due to decreased capacity for insulin secretion (7). However, metabolically favorable adiposity variants are linked to greater insulin sensitivity rather than greater insulin secretion (10). As insulin has been shown to act as a growth factor *in utero* (12), a possible mechanism for the effect of fetal metabolically favorable adiposity alleles on higher birth weight may be greater insulin sensitivity, allowing for a greater growth response to insulin secretion. It is also possible that fetal metabolically favorable adiposity alleles allow for greater fetal fat mass accumulation and hence greater birth weight. This possibility is supported by the fact that several of the metabolically favorable adiposity loci, in particular *PPARG* locus, have previously been found to be associated with adipocyte differentiation (19). Our analyses of cord-blood insulin and perinatal anthropometric traits aimed to assess evidence that higher fetal insulin secretion and/or fat mass might underlie the birth weight effects, but further studies with larger samples are needed to provide sufficient statistical power.

### Strengths and limitations

This study is the first to investigate the fetal effects of the recently discovered metabolically favorable adiposity associated genetic variants on fetal outcomes independent of the corresponding maternal genetic effects. It is also the largest study to investigate the effects of BMI associated genetic variants on fetal outcomes. To do this we attempted to use all available relevant data from multiple independent cohorts, to maximize the certainty of our findings.

Despite using all available mother–child cohorts with relevant data, our analyses had relatively low statistical power for outcomes other than birth weight, hence there is a need for larger cohort studies and/or GWAS of other perinatal outcomes.

There was substantial overlap between the samples used to identify the metabolically favorable adiposity and birth weight SNPs, which could bias our estimates due to statistical overfitting. To further investigate the possibility that the associations between metabolically favorable adiposity SNPs and birth weight might be inflated due to selection of the metabolically favorable adiposity SNPs from UK Biobank, we performed an additional analysis with BMI SNPs identified using only UK Biobank. Results were consistent between these analyses using BMI SNPs from UK Biobank to the main analyses excluding UK Biobank data. Whilst we cannot rule it out, these findings suggest that statistical overfitting is unlikely to have had a major impact on the result for metabolically favorable adiposity.

A further limitation to this study is the low response rate for UK Biobank (5.5%) (20) and the self-report of own birth weight in UK Biobank and some of the other studies included in EGG. A highly selected cohort such as UK Biobank can result in selection bias in genetic analyses (21,22). However, whilst self-report of birth weight is likely to have some measurement error, it is unlikely to affect the fetal genotype itself. The fact that the results for birth weight in EGG + UK Biobank were consistent with the results for birth weight of the four mother-offspring pair cohorts combined suggests that this bias is unlikely to have materially affected the results. For this study, we limited ourselves to European ancestry individuals, though relevant studies involving African ancestry individuals have been published (23), and additional studies involving non-European populations will be important going forward.

We have described the results of adiposity related genetic variants influencing birth weight, as the genetic variants cannot be influenced by the confounding of many social and environmental factors that confound conventional associations of non-genetic factors with outcomes, nor can genetic variants be influenced by existing disease. Our results were adjusted to avoid potential confounding by maternal genetic effects. We limited analyses to participants of European ancestry and the GWAS adjusted for principal components and center of recruitment, which should limit any confounding of the genetic effect due to population stratification. Whilst we do not think these findings are confounded, we acknowledge that we know little about the function of the adult adiposity related SNPs that we have shown here to influence birth weight.

In conclusion, our results suggest that fetal genetic predisposition to both higher metabolically favorable adult adiposity and higher general adiposity (proxied by BMI) result in higher birth weight. The effects of the BMI SNPs on birth weight are heterogeneous, with many of the strongest BMI associated SNPs showing no effect on birth weight. In contrast, metabolically favorable adiposity SNP effects on birth weight are more consistent, with those with larger effects on adult adiposity tending to have larger associations with birth weight. Larger population samples are needed to investigate the effects on other birth anthropometric outcomes and cord-blood markers, in order to elucidate the mechanisms underlying these effects.

## Materials and Methods

The aim of this genetic association study was to explore whether fetuses with a genetic predisposition to higher adult metabolically favorable adiposity or higher adult BMI also tend to have higher birth weights. This is based on our hypothesis that such genetic predisposition could result in faster fetal growth and hence higher birth weight.

We limited all analyses to participants of European ancestry, as genetic variants related to metabolically favorable adiposity and BMI were identified in GWAS of European ancestry individuals. We adjusted all analyses for maternal genotype effects to avoid confounding, since maternal and offspring genotypes are correlated and maternal genetic variants related to metabolically favorable adiposity or BMI are known to influence offspring birth weight (7,18,24).

### Main analyses: birth weight

**Figure 5:**
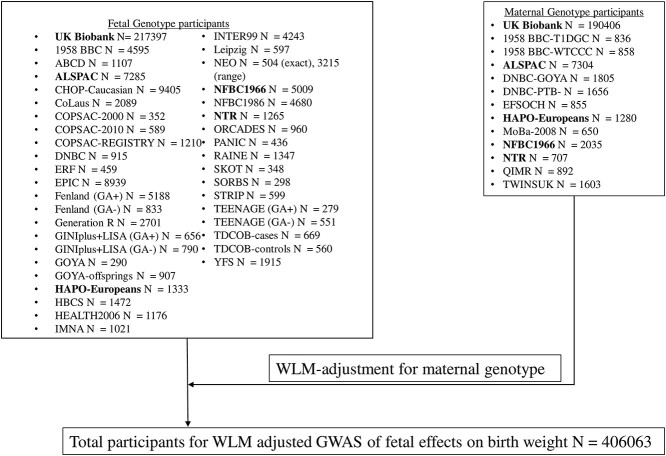
Outline of how all studies in the EGG+UK Biobank meta-analysis contributed to the final GWAS of fetal effects on birth weight (7). (1) Studies in bold contributed to both fetal and maternal genotype analyses.

Our analyses of genetic effects on birth weight used publicly available EGG consortium + UK Biobank summary GWAS data (see Fig. 5 for details on contributing studies). Summary data estimates of the fetal effects of selected SNPs on birth weight were combined into pooled genetic effects for metabolically favorable adiposity, and for BMI (details of pooling methods below). The fetal effects were adjusted for maternal genetic effects using a weighted linear model (WLM, see Supplementary Methods for further details) (7,25).

#### Data sources

##### EGG + UK Biobank

The EGG consortium component of the GWAS data for birth weight used in this study includes a meta-analysis of 35 studies of fetal genotype with birth weight (*N* = 80 745, European ancestry) as well as 12 studies of maternal genotype with offspring birth weight (*N* = 19 861, European ancestry), with some of the fetal and maternal studies overlapping (7). These studies were further meta-analyzed with summary data on European ancestry participants from the UK Biobank, which made up ~70% of the meta-analyses (with *N* = 217 397 contributing to fetal genotype analyses and *N* = 190 406 contributing to maternal genotype analyses). Between 2006 and 2010, UK Biobank participants were recruited from the NHS patient registers and contacted if they lived in close proximity to one of 22 assessment centers in England, Scotland and Wales. Detailed medical data was collected on 502 655 participants (5.5% response), aged between 40 and 69 at recruitment (26). All participants provided written informed consent, including for their collected data to be used by international scientists. UK Biobank has approval from the North West Multi-centre Research Ethics Committee (MREC), which covers the UK. UK Biobank’s research ethics committee and Human Tissue Authority research tissue bank approvals mean that researchers wishing to use the resource for approved health research do not need separate ethics approval.

In the EGG + UK Biobank birth weight GWAS (7), multiple births and preterm births were excluded. As gestational age is not available in UK Biobank, an approximation to excluding preterm births was achieved by excluding all births less than 2.2 kg. After excluding twins and inconsistently reported birth weight (difference of 0.5 kg between measures) only 5% of the population sample was lost due to excluding births less than 2.2 kg, which is unlikely to introduce collider bias into the population sample. Estimates for the fetal genetic effect on birth weight at each SNP was adjusted for the corresponding maternal genetic effect (see below for an explanation of how this was done), in addition to ancestry principal components and center of recruitment.

As UK Biobank (the largest contributing study to the GWAS) does not have sufficiently powered data on maternal–offspring pairs, the authors of that study (7) derived estimates of the fetal genetic effect on birth weight adjusted for the maternal genotype using a novel method which exploited the reporting by UK Biobank participants of their own birth weight (female and male participants) and the birth weight of their first born offspring (female participants only) (25). We refer to this as the WLM (details in Supplementary Methods). In total, the WLM–GWAS of fetal genotype on birth weight adjusted for maternal genotype used 406 063 participants (101 541 from UK Biobank with own and offspring birth weight, 195 815 from UK Biobank and EGG with own birth weight only and 108 707 from UK Biobank and EGG with maternal genotype and offspring birth weight only; see Fig. 5 for more details on the participants) (7). From the WLM–GWAS, we extracted the estimated fetal per-allele mean difference in birth weight and associated standard error (adjusted for the corresponding maternal effect) for each SNP independently with metabolically favorable adult adiposity (*N* = 14 SNPs) (10) and adult BMI (*N* = 76 SNPs) (8) at genome-wide *P*-value threshold (*P* < 5e-08).

#### Data analyses

##### Selecting genetic variants for analyses

The GWAS of metabolically favorable adult adiposity (*N* = 442 278) analyzed a composite phenotype characterized by increased body fat percentage and a metabolic profile related to a lower risk of type 2 diabetes, hypertension and heart disease, identified using multivariate GWAS (27) (details in Supplementary Methods) (10). Associations at a total of 14 loci were identified (at *P* < 5 × 10^−8^), each marked by a SNP at which one allele was associated with higher adult body fat percentage and a ‘favorable’ metabolic profile (10). We selected these 14 SNPs for our analyses. As these genetic variants were discovered using UK Biobank, and the birth weight GWAS used UK Biobank, there is sample overlap and a potential risk of overfitting and biasing the result towards a confounded association (28). To try and minimize this in the scatter plot analyses (see below), for each SNP we extracted the effect estimate from the most recent non-UK Biobank GWAS of body fat percentage (14).

For BMI, 76 SNPs were identified from the most recent European ancestry GWAS completed prior to the inclusion of the UK Biobank (*N* = 322 154) (8). Although 77 SNPs were reported in the GWAS (8), one SNP, rs7903146, in *TCF7L2*, is robustly associated with type 2 diabetes (29), and its association with BMI is in part likely due to collider bias, hence it was excluded.

As an additional sensitivity analyses, we checked to make sure that none of the 14 metabolically favorable adiposity SNPs were in strong pairwise LD with any of 76 BMI SNPs. Of the 14 metabolically favorable adiposity SNPs, only one was within a 1 Mb distance of a BMI SNP. Using LDlink and CEU as the reference population (30), we confirmed that none of the metabolically favorable adiposity SNPs was in strong pairwise LD (no pair had an *R*^2^ > 0.05) with any of the adult BMI SNPs.

##### Combining effects of individual SNPs on birth weight using summary data

To maximize power, we used random effects meta-analysis to combine the effects of multiple SNPs (i.e. 14 metabolically favorable adult adiposity SNPs or 76 adult BMI SNPs) on birth weight. This procedure allowed us to estimate the average effect of SNPs (for metabolically favorable adiposity and BMI) on birth outcomes without requiring individual level data. To do this, we first harmonized SNP-birth weight effect estimates for each SNP so that the effect allele would correspond to the allele increasing the adult adiposity trait (31). Then, we used random effects meta-analysis to combine SNP-birth weight effect estimates across SNPs, for the 14 favorable adiposity SNPs and the 76 BMI SNPs separately. For the main analyses, we used the EGG + UK Biobank summary results for the fetal effect on birth weight adjusted for maternal genotype (details in Supplementary Methods). We refer to the results as ‘pooled genetic effects’.

For the EGG + UK Biobank estimates, we converted the results back to grams by multiplying by 484 (the average SD for birth weight in grams for 18 studies in an early birth weight GWAS (32)).

### Scatter plot analyses

Using the summary GWAS data from the main analyses, we explored the plausibility of the *a priori* assumption that metabolically favorable adult adiposity and BMI alleles have effects on birth weight proportional to their effects on adult adiposity. To do this, we plotted scatter plots of the fetal effect estimate of each of the SNPs on birth weight (*y*-axis) (7) and on adult adiposity traits (either body fat percentage or BMI; *x*-axis) (8,14). We fitted a slope for each model using weighted linear regression, where we multiplied the SNP-adult trait estimate by the inverse of the standard error for the SNP-birth weight effect. As well as estimating the slope and intercept of the model, we also estimated the *R*^2^ value as a measure of variance explained by adult adiposity (33). This approach also allowed us to visually inspect for the presence of outliers.

### Exploratory analyses: birth anthropometric measures and cord-blood outcomes

**Figure 6:**
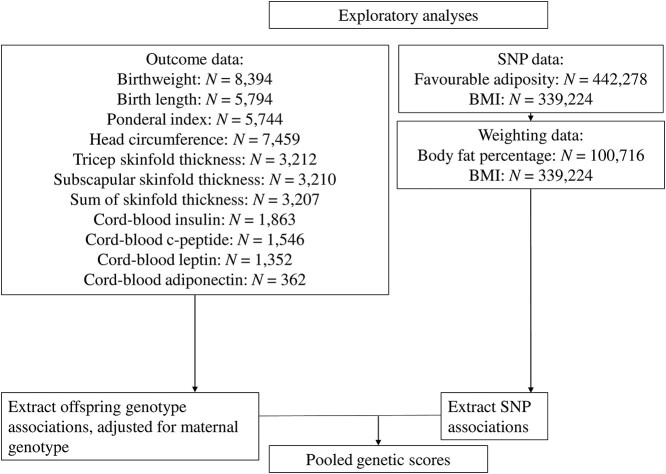
Outline of all studies that contributed to the exploratory analyses.

To further understand the associations between fetal genetic variants and components of birth weight or related cord-blood phenotypes, we performed exploratory analyses of other birth outcomes using individual level data from four birth cohorts (see Fig. 6, Supplementary Material, Tables S1 and S2 for more details), each analyzed separately with results pooled using fixed effect meta-analysis. As these analyses were exploratory, we did not correct the results for multiple testing. The analyses were adjusted for the maternal genetic effects using direct adjustment for the maternal genotype.

#### Data sources

##### ALSPAC

The Avon Longitudinal Study of Parents and Children (ALSPAC) is a birth cohort that recruited 14 541 pregnancies to women who were resident in and around the city of Bristol in the South West of the UK and who had expected dates of delivery between 1 April 1991 and 31 December 1992 (34,35). Of these 14 541, there were known live birth outcomes in 13 867 pregnancies to 13 761 women. Please note that the study website contains details of all the data that are available through a fully searchable data dictionary and variable search tool (36). We used principal components from Hapmap II (release 22) to separate out European ancestry in the genotyped individuals. Maternal genomic data were obtained from the Illumina Human610 Quad Array and fetal data were obtained from the Illumina HumanHap550 Quad Array. The genotypes were imputed using the Haplotype Reference Consortium HRC v1.1 reference panel after quality control (MAF > 1%, HWE > 1 × 10^−7^, sex mismatch and kinship errors). In this study, we used a maximum of 4862 unrelated and genotyped mother–child pairs with phenotype data. This cohort contributed to the analyses with the following outcomes: birth weight (included also in the EGG consortium GWAS of birth weight), birth length, birth ponderal index and birth head circumference. Mothers provided written informed consent and ethical approval for the study was obtained from the ALSPAC Ethics and Law Committee and the Local Research Ethics Committees.

##### BiB

Born in Bradford (BiB) is a population-based prospective pregnancy cohort that collected detailed information from 12 450 women who experienced 13 773 pregnancies (37). The cohort is broadly representative of the obstetric population in Bradford, a city in the North of England, in which approximately half of the births are to mothers of South Asian origin. To be eligible for BiB, women had to have an expected delivery date between March 2007 and December 2010 in the maternity department of the Bradford Royal Infirmary. Participants were recruited primarily at their oral glucose tolerance test (OGTT) appointment, mostly between 26 and 28 weeks. BiB is a multi-ethnic cohort of mostly White Europeans and South Asians. Ethnicity was based on self-report for most participants, and where that self-report was unavailable, ethnicity reported in general practitioner (GP) records was used. For a small number of participants where neither self-report nor GP records of ethnicity, South Asian ethnicity was defined using Nam Pechan (37), a computer program for identifying South Asian names (38), and those not identified as South Asian by Nam Pechan were assumed to be White British and included in this study. Maternal and fetal genomic data were obtained from two separate chips, an Illumina HumanCoreExome array and Illumina Infinium Global Screening array (GSA), and genotype data were imputed against HRC r1.1 using Minimac4, after quality control (MAF > 1% and HWE > 1 × 10^−6^). In this study, we used a maximum of 1947 unrelated and European ancestry genotyped mother–child pairs with phenotype data. This cohort contributed to the analyses with the following outcomes: birth weight, birth head circumference, birth triceps skinfold thickness, birth subscapular skinfold thickness, sum of skinfold thickness, maternal fasting glucose, maternal 2 h post-prandial glucose levels, cord-blood insulin, cord-blood leptin (a marker of fetal fat mass (39)) and cord-blood adiponectin. Cord blood was extracted from a vein or artery by the attendant mid-wife at delivery. Samples were refrigerated at 4°C in EDTA tubes until collected by laboratory staff within 12 h. Samples were then spun, frozen and stored at −80°C. They were transferred to the Biochemistry Department of Glasgow Royal Infirmary for analyses (with no previous thawing), where leptin and adiponectin were measured by a highly sensitive in house ELISA with better sensitivity at lower levels than commercial assays. Insulin was measured using an ultrasensitive solid-phase two-site immunoassay ELISA (Mercodia, Uppsala, Sweden) that does not cross-react with pro-insulin (40). Laboratory staffs were blinded to the participants ethnicity and other characteristics. Ethics approval was obtained for the main platform study and all of the individual sub-studies from the Bradford Research Ethics Committee (37).

##### EFSOCH

The Exeter Family Study of Childhood Health (EFSOCH) is a birth cohort that recruited 1017 families who were resident in the postcode-defined area of central Exeter between 2000 and 2004, at the Royal Devon and Exeter Hospital (41) from which a total of 993 live births were included in the analyses of this paper. Maternal and fetal genomic data were obtained from the Illumina Infinium HumanCoreExome-24, and the genotypes were imputed against Haplotype Reference Consortium HRC v1.1 reference panel after quality control (MAF > 1%, HWE > 1 × 10^−6^, sex mismatch, kinship errors and 4.56 SD from the cluster mean of any sub-populations cluster). In this study we used a maximum of 674 unrelated and genotyped mother–child pairs with phenotype data. This cohort contributed to the analyses with the following outcomes: birth weight (included also in the EGG consortium GWAS of birth weight), birth length, birth ponderal index, birth head circumference, birth triceps skinfold thickness, birth subscapular skinfold thickness, sum of skinfold thickness, maternal fasting glucose and cord-blood insulin. Cord blood was extracted from a vein or artery by the attendant mid-wife at delivery. The blood was stored at 4°C until being collected by the researchers. The cord blood was spun to separate out the plasma which was then stored at −80°C. The plasma was then tested for insulin levels when appropriate at the Regional Endocrine Laboratories (Birmingham, UK) using immunochemiluminometric assays (Molecular Light Technology, Cardiff, UK) (41,42). All mothers and fathers gave informed consent and ethical approval was obtained from the local review committee.

##### HAPO

The Hyperglycemia and Adverse Pregnancy Outcome (HAPO) cohort recruited 28 562 pregnant women between 1 July 2000 and 30 April 2006 from 15 clinical study centers in 10 countries (United States, Canada, Barbados, United Kingdom, the Netherlands, Thailand, Israel, Australia, Hong Kong and Singapore), four of the centers being in the United States, for their oral glucose tolerance test (OGTT) between 24 and 32 weeks (43). In total, 25 505 pregnant women underwent OGTT, however only 23 316 women were blind tested (participants were un-blinded if they showed signs of having diabetes, i.e. fasting plasma glucose > 5.8 mmol/l or 2 h glucose > 11.1 mmol/l). The protocol was approved by the institutional review board at each field center. HAPO is a multi-ethnic cohort, and ethnicity was self-reported by the participants (43). Maternal and fetal genomic data was obtained from Illumina genome-wide arrays at the Broad Institute (Cambridge, MA) or Johns Hopkins Center for Inherited Disease Research (Baltimore, MD). The genotypes were imputed using SHAPEIT v.2 and IMPUTE2 v.2.3.0 with 1000 Genomes Phase 3 data after quality control as previously described. In this study, we used a maximum of 1867 unrelated and European ancestry genotyped mother–child pairs with phenotype data. This cohort contributed to the analyses with the following outcomes: birth weight (included also in the EGG consortium GWAS of birth weight), birth length, birth ponderal index, birth head circumference, birth triceps skinfold thickness, birth subscapular skinfold thickness, sum of skinfold thickness, maternal fasting glucose, maternal 2 h post-prandial glucose levels and cord-blood c-peptide. Cord-blood plasma was extracted at each center, was stored at −20°C and sent to the Central Laboratory for analysis. A subset of plasma was then stored at −70°C, before being tested for c-peptide using a solid-phase, two-site fluoro-immunometric assay (Autodelfia, Perkin-Elmer, Waltham, Massachusetts, United States). C-peptide has an advantage over insulin in that it is less likely to be destroyed by hemolysis, thus allowing for a more accurate representation of cord insulin levels if hemolysis has occurred in a substantial number of samples (44). All participants gave written informed consent. An external data and safety monitoring committee provided oversight.

#### Data Analyses

We selected the genetic variants for analyses and combined the effects of individual SNPs on perinatal outcomes the same way we did for the main analyses (see Main analyses: birth weight: Data Analyses for more details). Birth weight and all other perinatal traits were standardized within each cohort separately when estimating the metabolically favorable adiposity and BMI pooled genetic effects, in order to make the estimates for each trait comparable with each other.

##### Estimating effects of individual genetic variants on birth anthropometric and cord-blood markers in the four birth cohorts

Within each of the four cohorts, we analyzed individual level data using linear regression of birth weight or other perinatal traits on the SNPs (adjusting for gestational age, the child’s sex and maternal genotype). Linear regression analyses were performed separately in each cohort and the results pooled using fixed-effects meta-analysis, resulting in summary data on the association between each SNP and each phenotype. Further information on these cohorts and their contribution to the study can be found in Supplementary Material, Table S1 and details on how the anthropometric outcomes were measured can be found in Supplementary Material, Table S2.

Though our analyses of birth weight and other perinatal traits was restricted to European ancestry participants, the cohorts used came from different locations and used different methods to measure perinatal traits. To test for between study heterogeneity, we performed Cochran’s *Q* test and estimated *I*^2^ for each meta-analyses (45).

### Sensitivity analysis using BMI SNPs identified using UK Biobank

Metabolically favorable adiposity SNPs were selected from UK Biobank, whilst the BMI SNPs were not. As an additional analyses, to assess whether this could bias the metabolically favorable adiposity association estimates away from the null, we also selected SNPs associated with BMI in UK Biobank, and extract SNP-adult adiposity effect estimates for them from a UK Biobank-independent source, as we had done with metabolically favorable adiposity. There were 458 SNPs identified from a recent UK Biobank GWAS of BMI (*N* = 461 460) (15), and of those, we were able to extract weights for a total of 393 SNPs (*n* = 210 lead SNPs and a further 183 close (*R*^2^ > 0.8) proxies) from the GIANT consortium data set (8). We excluded one SNP, which was in strong pairwise LD (*R*^2^ > 0.4) with a metabolically favorable adiposity SNP, leaving a total of 392 SNPs.

We performed the same analyses for the 392 SNPs associated with BMI identified in UK Biobank as we did for the 76 SNPs identified in the GIANT consortium (see Main analyses, Data analyses, Combining effects of individual SNPs on birth weight using summary data and Scatter plot analyses).

## Data Availability

Our study used both published summary results (i.e. taking results from published research papers and websites) and individual participant cohort data as follows:

The data for the GWAS of BMI are available here.

https://portals.broadinstitute.org/collaboration/giant/index.php/GIANT_consortium_data_files

The data for the GWAS of body fat percentage are available here.

https://walker05.u.hpc.mssm.edu

The data for the GWAS of birth weight are available here.

https://egg-consortium.org/birth-weight-2019.htm

The references to those published data sources are provided in the main paper.

We used individual participant data for the genetic association analyses from the UK Biobank, ALSPAC, BiB, EFSOCH and HAPO cohorts.

The data in UK Biobank, ALSPAC and BiB are fully available, via managed systems, to any researchers. The managed system for both studies is a requirement of the study funders but access is not restricted on the basis of overlap with other applications to use the data or on the basis of peer review of the proposed science.

UK Biobank. Full information on how to access these data can be found here—https://www.ukbiobank.ac.uk/using-the-resource/

ALSPAC. The ALSPAC data management plan (http://www.bristol.ac.uk/alspac/researchers/data-access/documents/alspac-data-management-plan.pdf) describes in detail the policy regarding data sharing, which is through a system of managed open access. The steps below highlight how to apply for access to the data included in this paper and all other ALSPAC data.

1. Please read the ALSPAC access policy (PDF, 627kB) that describes the process of accessing the data and samples in detail, and outlines the costs associated with doing so.
2. You may also find it useful to browse the fully searchable ALSPAC research proposals database, which lists all research projects that have been approved since April 2011.
3. Please submit your research proposal for consideration by the ALSPAC Executive Committee. You will receive a response within 10 working days to advise you whether your proposal has been approved.

If you have any questions about accessing data, please email alspac-data@bristol.ac.uk.

BiB. Full information on how to access these data can be found here—https://borninbradford.nhs.uk/research/how-to-access-data/

HAPO. For access to the data used in this study, please contact Dr Rachel Freathy (r.freathy@ex.ac.uk) and Prof. William Lowe Jr (wlowe@northwestern.edu). The website describing the study and other data available is https://www.ncbi.nlm.nih.gov/projects/gap/cgi-bin/study.cgi?study_id=phs000096.v4.p1

If you have further questions, please email Dr William Lowe at wlowe@northwestern.edu

EFSOCH. Requests for access to the original EFSOCH dataset should be made in writing in the first instance to the EFSOCH data team via the Exeter Clinical Research Facility crf@exeter.ac.uk.

## Supplementary Material

Supplementary_Material_for_infants_own_genetic_propensity_for_resubmission2_ddab356
