# Supplementary material for: Fetal alleles predisposing to metabolically favorable adiposity are associated with higher birth weight
Source: Hum Mol Genet. 2021 Dec 13;31(11):1762–75. doi: 10.1093/hmg/ddab356 (PMC9169452; doi:10.1093/hmg/ddab356)
Supplement: Supplementary_Material_for_infants_own_genetic_propensity_for_resubmission2_ddab356 [file supplementary_material_for_infants_own_genetic_propensity_for_resubmission2_ddab356.docx]

**Supplementary Material**

Table of Contents

[**Supplementary Methods** 2](#_Toc85816178)

[**Using Structural Equation Modelling (SEM) to estimate maternal and fetal genetic effects on birth weight** 2](#_Toc85816179)

[**Deriving the metabolically favourable adiposity phenotype and identifying genetic variants related to this phenotype** 3](#_Toc85816180)

[**Supplementary Tables** 5](#_Toc85816181)

[**sTable 1: Characteristics of the studies used for analyses of all birth outcomes** 5](#_Toc85816182)

[**sTable 2: Measurement of birth anthropometric traits in selected cohorts** 6](#_Toc85816183)

[**References** 7](#_Toc85816184)

# **Supplementary Methods**

## **Using Structural Equation Modelling (SEM) to estimate maternal and fetal genetic effects on birth weight**

SEM can be used to estimate the fetal genetic effect on own birth weight conditional on maternal genotype in the absence of data from genotyped mother-child pairs(1). The model uses the participant’s own genotype, own birth weight and their offspring’s birth weight as observed data. For each individual, these observed variables are modelled as functions of two latent (unobserved) variables, the individual’s mother’s genotype and the individual’s offspring’s genotype, which are both correlated at a level of 0.5 with the participant’s own genotype. A full description of the structural equation model can be found in Warrington et al 2018(1). In brief, the model uses the variances and co-variances between the observed variables (own birth weight, offspring birth weight and own genotype), to estimate the parameters of interest including fetal and maternal genetic effects on birth weight. The model is flexible in that it can incorporate a subset of participants with only their own birth weight and own genotype as well as a subset of participant’s with only their own genotype and offspring birth weight. However, fitting the model using full information maximum likelihood is computationally intensive, making it difficult to use at a genome-wide level. Consequently, the authors developed a linear approximation of the full SEM that yielded similar effect estimates and standard errors but was more computationally efficient. This linear approximation, referred to as the weighted linear model (WLM) approximation, combined unadjusted fetal and maternal genetic effect estimates at a single locus, using the following formula

$$\hat{\beta}_{f_{adj}}= -\frac{2}{3}\hat{\beta}_{m_{unadj}}+\frac{4}{3}\hat{\beta}_{f_{unadj}}$$

where $\hat{\beta}_{f_{adj}}$ is the estimated fetal genetic effect (adjusted for maternal genotype)on the outcome (in this case birth weight), $\hat{\beta}_{m_{unadj}}$ is the unadjusted maternal genetic effect from an unconditional GWAS of maternal genotype and offspring birth weight and $\hat{\beta}_{f_{unadj}}$ is the unadjusted fetal genetic effect from an unconditional GWAS of fetal genotype and own birth weight (1, 2)

Standard errors for the fetal genetic effect (adjusted for maternal genotype) can be calculated, assuming no overlapping individuals across the samples, using the following formula.

$$SE\left( \hat{\beta}_{f_{adj}} \right)= \sqrt{\left( \frac{4}{9}var\left( \hat{\beta}_{m_{unadj}} \right)+\frac{16}{9}var\left( \hat{\beta}_{f_{unadj}} \right) \right)}$$

The estimated fetal genetic effect from the WLM,$\hat{\beta}_{f_{adj}}$ , has been shown to be asymptotically unbiased and similar to the estimated effect from a conditional linear model in mother-child pairs, where own birth weight is regressed on own genotype and maternal genotype(2).

## **Deriving the metabolically favourable adiposity phenotype and identifying genetic variants related to this phenotype**

The metabolically favourable adult adiposity genetic variants were identified in a previous study(3) in three steps. In step 1 a GWAS for body fat percentage, as measured by bioimpedance, was performed in the UK Biobank (N = 442,278)(3).

In step 2, a multivariate GWAS was performed combing several metabolic biomarkers together in the same multivariate analyses (i.e. body fat percentage, high density lipoprotein (HDL) cholesterol, adiponectin, sex-hormone binding globulin, triglycerides, fasting insulin and alanine transferase). In order to perform a multivariate GWAS, canonical correlation analyses were conducted as implemented by the metaCCA package in R(4).

Standard univariate GWAS analyses of quantitative traits use linear regression to estimate the linear relationship between one SNP at a time and a single outcome of interest (sometimes adjusting for covariates in the multivariable regression model).

Canonical correlation analyses on the other hand involves estimating the maximum correlation between an optimally weighted linear combination of exposure variables and an optimally weighted linear combination of outcome variables. When used in the context of GWAS, this allows one to see how a given genetic variant associates with a linear combination of observed traits, in this particular instance, a combination of traits which index a metabolically favourable vs unfavourable profile.

Traditionally, in order to perform multivariate GWAS using canonical correlation analyses, individual level participant data would be needed. However, the metaCCA program allows one to perform canonical correlation analyses using summary results data(4).

In step 3, SNPs that were associated at p < 5 x 10^-8^ with both higher body fat percentage (step 1) and with a metabolically favourable profile in the multivariate GWAS of metabolic traits (step 2) were selected. This was achieved using hierarchical clustering using the pvclust R package, a method which groups of genetic variants are clustered based on their differing associations with observed phenotypes. Finally, the genetic variants identified were then replicated in five obesity cohorts(3).

# **Supplementary Tables**

|  | ALSPAC | BiB | EFSOCH | HAPO 1^a^ | HAPO 2^a^ |
| --- | --- | --- | --- | --- | --- |
| Number of Mother-Offspring pairs | 7411 | 3308 | 1022 | 1052 | 815 |
| Country | United Kingdom | United Kingdom | United Kingdom | United States | United States |
| Offspring years of birth | 1991-1993 | 2007-2011 | 2000-2004 | 2001-2006 | 2000-2006 |
| Maternal Age at birth of child (years) | 28.5 (4.8) | 27.1 (6) | 30.4 (5.3) | 32.1 (5.1) | 29.9 (5.4) |
| Maternal pre-pregnancy BMI (kg/m2) | 22.9 (3.8) | 26.6 (5.9) | 24 (4.4) | 24.2 (4.6) | 24.6 (5.3) |
| Gestational age at delivery (weeks) | 39.6 (1.7) | 39.7 (1.8) | 39.9 (1.5) | 40 (1.2) | 40 (1.2) |
| Offspring sex (% male) | 49.8 | 51.6 | 51.6 | 47.9 | 50.9 |
| Mothers smoking (%) | 17.2 | 33.1 | 13.3 | 12.9 | 15.1 |
| Birth Weight (g) | 3495 (471) | 3439 (482) | 3513 (476) | 3543 (509) | 3540 (431) |
| Birth Length (cm) | 50.9 (2.2) | NA | 50.3 (2.1) | 50.5 (2.2) | 51.8 (2.5) |
| Birth Ponderal Index (kg/m3) | 26.4 (2.7) | NA | 27.7 (2.6) | 27.4 (3.3) | 25.4 (3.3) |
| Birth Head Circumference (cm) | 35 (1.4) | 34.7 (1.4) | 35.2 (1.3) | 34.9 (1.6) | 34.9 (1.4) |
| Birth Triceps Skinfolds (mm) | NA | 5.2 (1.1) | 4.9 (1.1) | 4.1 (0.8) | 4.1 (0.9) |
| Birth Subscapular Skinfolds (mm) | NA | 4.9 (1.1) | 4.9 (1.2) | 4.6 (1) | 4.3 (1) |
| Sum of Birth Skinfolds (mm) | NA | 10.1 (2.1) | 9.7 (2.1) | 13.1 (2.5) | 12.3 (2.4) |
| Cord-blood C-Peptide (μg/mL)^b^ | NA | NA | NA | 0.9 (0.7-1.3) | 0.9 (0.7-1.3) |
| Cord-blood insulin (pg/mL)^b^ | NA | 3.5 (2.1-5.8) | 37.6 (26-60) | NA | NA |
| Cord-blood leptin (ng/mL)^b^ | NA | 7.3 (4-13.1) | NA | NA | NA |
| Cord-blood adiponectin (μg/ml)^b^ | NA | 33.3 (26.3-42.7) | NA | NA | NA |
| Fasting Glucose (mmol/l) | NA | 4.4 (0.42) | 4.35 (0.38) | 4.58 (0.37) | 4.51 (0.34) |
| 2 hour postload glucose (mmol/l) | NA | 5.43 (1.3) | NA | 6.02 (1.2) | 6.06 (1.19) |

## **sTable 1: Characteristics of the studies used for analyses of all birth outcomes**

1. For HAPO 1, genetic data was stored and analysed at the Northwestern University Feinberg School of Medicine, Chicago. For HAPO 2, genetic data was stored and analysed at the University of Exeter. These were non-overlapping samples of European mothers and babies.
2. For the cord-blood outcomes, because they have a non-standard distribution, the median and interquartile range are displayed. For all other outcomes, the mean value and the standard deviation are displayed.

## **sTable 2: Measurement of birth anthropometric traits in selected cohorts**

| Trait | ALSPAC(5, 6) | BiB(7, 8) | EFSOCH(9) | HAPO(10) |
| --- | --- | --- | --- | --- |
| Birth Weight | Extracted from clinical records | Extracted from clinical records | Calibrated electronic scale | Calibrated electronic scale |
| Birth Length | Extracted from clinical records | NA | Standardized plastic board | Standardized plastic board |
| Ponderal Index | Calculated | NA | Calculated | Calculated |
| Head Circumference | Extracted from clinical records | Standardized measuring tape | Standardized measuring tape | Standardized measuring tape |
| Triceps Skinfold | NA | Calipers | Calipers | Calipers |
| Subscapular Skinfold | NA | Calipers | Calipers | Calipers |
| Sum of Skinfolds | NA | Calculated | Calculated | Calculated |

# **References**

1 Warrington N.M., Freathy R.M., Neale M.C. and Evans D.M. (2018) Using structural equation modelling to jointly estimate maternal and fetal effects on birthweight in the UK Biobank. *Int. J. Epidemiol.*, in press., 1229-1241.

2 Warrington N.M., Beaumont R.N., Horikoshi M., Day F.R., Helgeland Ø., Laurin C., Bacelis J., Peng S., Hao K., Feenstra B. *et al.* (2019) Maternal and fetal genetic effects on birth weight and their relevance to cardio-metabolic risk factors. *Nat. Genet.*, **51**, 804-814.

3 Ji Y., Yiorkas A.M., Frau F., Mook-Kanamori D., Staiger H., Thomas E.L., Atabaki-Pasdar N., Campbell A., Tyrrell J., Jones S.E. *et al.* (2019) Genome-Wide and Abdominal MRI Data Provide Evidence That a Genetically Determined Favorable Adiposity Phenotype Is Characterized by Lower Ectopic Liver Fat and Lower Risk of Type 2 Diabetes, Heart Disease, and Hypertension. *Diabetes*, **68**, 207-219.

4 Cichonska A., Rousu J., Marttinen P., Kangas A.J., Soininen P., Lehtimäki T., Raitakari O.T., Järvelin M.-R., Salomaa V., Ala-Korpela M. *et al.* (2016) metaCCA: summary statistics-based multivariate meta-analysis of genome-wide association studies using canonical correlation analysis. *Bioinformatics*, **32**, 1981-1989.

5 Fraser A., Macdonald-Wallis C., Tilling K., Boyd A., Golding J., Davey Smith G., Henderson J., Macleod J., Molloy L., Ness A. *et al.* (2013) Cohort Profile, the Avon Longitudinal Study of Parents and Children: ALSPAC mothers cohort. *Int. J. Epidemiol.*, **42**, 97-110.

6 Boyd A., Golding J., Macleod J., Lawlor D.A., Fraser A., Henderson J., Molloy L., Ness A., Ring S. and Davey Smith G. (2013) Cohort Profile, The ‘Children of the 90s’—the index offspring of the Avon Longitudinal Study of Parents and Children. *Int. J. Epidemiol.*, **42**, 111-127.

7 Mebrahtu T.F., Feltbower R.G. and Parslow R.C. (2015) Effects of birth weight and growth on childhood wheezing disorders, findings from the Born in Bradford Cohort. *BMJ. Open.*, **5**, e009553.

8 Wright J., Small N., Raynor P., Tuffnell D., Bhopal R., Cameron N., Fairley L., Lawlor D.A., Parslow R., Petherick E.S. *et al.* (2012) Cohort Profile, The Born in Bradford multi-ethnic family cohort study. *Int. J. Epidemiol.*, **42**, 978-991.

9 Knight B., Shields B.M. and Hattersley A.T. (2006) The Exeter Family Study of Childhood Health (EFSOCH), study protocol and methodology. *Paediatr. Perinat. Epidemiol.*, **20**, 172-179.

10 Group H.S.C.R. (2009) Hyperglycemia and Adverse Pregnancy Outcome (HAPO) Study, associations with neonatal anthropometrics. *Diabetes*, **58**, 453-459.
